# MicroRNA-497 Inhibits Cardiac Hypertrophy by Targeting Sirt4

**DOI:** 10.1371/journal.pone.0168078

**Published:** 2016-12-16

**Authors:** Yimin Xiao, Xiaofei Zhang, Shihao Fan, Guanghao Cui, Zhenya Shen

**Affiliations:** 1 Department of Cardiovascular Surgery of the First Affiliated Hospital & Institute for Cardiovascular Science, Soochow University, Suzhou, China; 2 Department of Cardiovascular Surgery, Shanghai Yodak Cardiothracic Hospital, Shanghai, China; University of Cincinnati College of Medicine, UNITED STATES

## Abstract

Cardiac hypertrophy is an adaptive enlargement of the myocardium in response to overload pressure of heart. From abundant studies, a conclusion is drawn that many microRNAs (miRNAs) are associated with cardiac hypertrophy and heart failure. To investigate the role of microRNA-497 (miR-497) in myocardial hypertrophy, two models were established in this study from cell level to integral level. Cardiac hypertrophy was induced by using angiotensin Ⅱ (Ang Ⅱ) in vitro and was created by transverse abdominal aortic constriction (TAC) in vivo. There was a significant decrease expression of miR-497 in cardiac hypertrophy models. Moreover, overexpression of miR-497 inhibited myocardial hypertrophy both in vitro and in vivo without heart function variation. In addition, luciferase reporter assays demonstrated that Sirt4 was a direct target gene of miR-497. Taking together, our study indicates that miR-497 modulates cardiac hypertrophy by targeting Sirt4 and may serve as a potential therapeutic substance in the course.

## Introduction

World Health Organization announced that a total number of 17,500,000 people die from heart failure each year, which account for 30% of the death case [1]. Cardiac hypertrophy is a major determinant for the development of heart failure and is associated with a higher risk of sudden cardiac death (SCD) [2]. Though cardiac hypertrophy is not a disease in essence, it’s a symptom of a serious condition which may place human’s life in great danger by evolving into heart failure under sustainable high pressure.

MiRNAs are endogenous, single-stranded, short non-coding RNAs that act as regulators of gene expression by promoting the degradation or inhibiting the translation of target mRNAs [3]. It is reported that miRNAs are involved in varieties of normal function of eukaryotic cells, including proliferation, apoptosis, development and so on [4]. Relationships between miRNAs dysregulation and human disease are investigated from variable disease. MiR-497, being one of miR-15 family members, is mainly identified as a tumor suppressor in various cancers, such as hepatocellular carcinoma [5], osteosarcoma [6–7] and breast cancer [8]. Recently, miR-15 family members were identified as novel regulators in cardiac hypertrophy and fibrosis by inhibition of the TGFβ pathway [9]. However the exact function of miR-497 in cardiomyocytes or heart remains uncertain. Our study is focused on the exact effect of miR-497 in cardiac hypertrophy, which may offer a new outlook for human being on it.

In this study, our experimental data demonstrated a direct myocardial role for miR-497 as a critical regulator in the development of cardiac hypertrophy and provided a useful model to elucidate its mechanisms in the pathogenesis of cardiac hypertrophy. We offered further proof for the first time that the overexpression of miR-497 significantly suppressed cardiac hypertrophy by inhibiting the expression of Sirt4. These findings disclosed a new connection between Sirt4 and miR-497 in the development of cardiac hypertrophy and failure.

## Materials and Methods

### Primary cardiomyocyte isolation and culture

Neonatal mouse cardiomyocytes were separately isolated from the hearts of 1–2-day-old newborn C57BL6 mice as described previously [10]. Isolated cardiomyocytes were seeded in pre-coated 24-well plates at 1.5×10^4^ cells per well in B/D medium (Life Technologies, Foster City, CA) containing 50% Dulbecco’s minimum essential medium (DMEM, Life Technologies) together with 10% fetal calve serum (FCS, Hyclone, Logan, UT, USA). The cells were cultivated in an incubator at 37°C in a humid atmosphere consisting of 5% CO2 and the cultures were kept in a semi-confluent condition to prevent cell from cellular differentiation. Furthermore, Bromodeoxyuridine of 0.1 mM was added to the culture media for the first 72 hours to further minimize contamination from fibroblasts. The elimination of fibroblasts and other proliferations was performed through preplating for 90 minutes at 37°C to achieve the great purity of cardiomyocytes.

### Animal model

All mice were purchased from Laboratory Animal Centre, Soochow University and were fed on autoclaved food in the pathogen-free animal room with access to the reasonable and controllable temperature, light and humidity. All experiments and animal care procedures were approved by Ethics Review Board of Soochow University and done following institutional guidelines. Firstly body weight was measured for individual mice. After mice were anesthetized with ketamine (100 mg/kg), TAC was carried out to induce the cardiac hypertrophy of mice. To be brief, a 4–0 suture was employed to tie two circles around the abdominal aorta by 21-gauge needle to decrease the outer aortic by 0.3 mm approximately, after which the needle was eliminated, while the sham group was subjected to the same cutting and stitching course as the experimental group in order to control variables. 8 weeks later, the mice were anesthetized with ketamine, and the cardiac dimensions and function were analyzed with 7.5-MHz pulse-wave Doppler echocardiography (Philips IE33). Then, the mice were killed by decapitation, and hearts were gathered for further evaluation.

### Histological analysis

First, the myocardial tissue samples were fixed by immersion in 4% paraformaldehyde for 24 to 48 h. Then, myocardial tissue was embedded in paraffin and stained with haematoxylin and eosin (H&E). The cardiomyocyte and paraffin-embedded tissue sections were incubated with a mouse monoclonal (α-actin) antibody (1:100, Abcam, LA, USA) for at 4°C overnight. An Alexa Fluor 594 goat anti-mouse antibody (1:200, Abcam, LA, USA) was used as a secondary antibody. DNA in the nucleus of cardiomyocytes was stained with 0.025 μg/ml DAPI at room temperature without any exposure to light. Finally, the slides were mounted with Vectashield and 4',6-diamidino-2-phenylindole mounting medium without being exposed to light at 4°C overnight, and fluorescently labelled cells were closely scrutinized using a fluoview 1000 confocal microscope (Olympus, Osaka, Japan). A random collection of 10 cardiomyocytes images was created for the calculation of cardiomyocyte area using Image J software. Quantification of fluorescence intensities was carried out using MetaMorph software (Boyce Scientific, St. Louis, MO).

### Recombinant lentivirus construction and infection

Both the primary miR-497 and negative control sequence were synthesized by Genepharma (Shanghai, China) inserted into the Pglv3/h1/GFP plasmid vector (Genepharma, Shanghai, China) to construct specific miRNA-overexpressing lentivirus. The negative control sequence is a random sequence that has been extensively tested in mouse cell lines and tissues and validated to not produce identifiable effects on known miRNA function. The pseudoviral particles were produced using a lentivector packaging system (Genepharma) according to the manufacturer's instructions. Cells were infected with the miR-497 lentivirus (Leti-miR-497) or negative control lentivirus (Leti-NC) at an MOI of 50. TAC mice were subject to chest reopening with injection of indicated lentivirus (3.5×10^7^ viral particles per mice). The infection efficiency was evaluated by observing GFP expression under a fluorescence microscope after 48 h.

### Real-time quantitative PCR (qRT-PCR) analysis

Total RNA from cardiomyocytes and myocardial tissue was isolated and obtained using Trizol (Invitrogen, Carlsbad, CA). Briefly, 2 μg of extracted RNA was reverse transcribed to cDNA with the application of reverse transcriptase (Takara, Tokyo, Japan). The expression levels were quantitatively analysed using a standard SYBR green PCR kit (Promega, Madison, WI, USA). GAPDH was used as an internal control. The primer sequences were as follows: MHC, forward: 5'-CCTCGCAATATCAAG GGAAA-3', reverse: 5'-TACAGGTGCATCAGCTCCAG-3'; ANP, forward: 5-GGGGGTAGGATTG ACAGGAT -3', reverse: 5'-CTCCAGGAGGGTATTCACCA-3'; GAPDH, forward: 5'-AAGAAGGTG GTGAAGCAGGC-3', reverse: 5'-TCCACCACCCAGTTGCTGTA-3'.

MiRNAs were quantified by using TaqMan MicroRNA Assays (Applied Biosystems, Foster City, CA). The first strand cDNA synthesis of each miRNA was performed through reverse transcription with use of TaqMan MicroRNA Reverse Transcription Kit (Applied Biosystems). U6 was applied as the internal control for target genes. The performance of analysis was performed by 2^−ΔΔCT^ method.

### Tritium-leucine incorporation assay

Neonatal cardiomyocytes were cultured on 24-well plates at a density of 4×10^5^ cells/ml. After the incubation with Ang Ⅱ, the cardiomyocytes were treated with 37 kBq tritium-leucine (China Institute of Atomic Energy, Beijing, China) with a final concentration of 3.7×10^4^ Bq/ml for 48 h. The incubation was finished by putting 100 μl of 50% TCA into the plates, followed by measurement of the incorporated tritium-leucine with the application of Packard Tri-Carb 2100TR Liquid Scintillation Analyzer (GMI, Inc, Ramsey, MN, USA).

### Western blot

Cardiomyocytes were collected and lysed after which equivalent quantity of protein was loaded and separated by 10% SDS-PAGE. Then all the separated protein was electrotransferred onto polyvinylidene fluoride (PVDF) membranes. The membrane was blocked by 5% skim milk for 1h, incubated with primary antibodies (Sirt4, 1:1000, Santa Cruz Biotechnology, Inc., Dallas, Texas, USA; GAPDH, 1:2000, Cell Signaling Technology, Boston, USA) at 4°C overnight. Afterwards horseradish peroxidase-conjugated secondary antibody (1:10,000, Abcam, LA, USA) was also incubated on the membrane at 37°C for 1 h. Proteins were scanned and detected by enhanced chemiluminescence (Bio-Rad Laboratories, Hercules, CA, USA) using a ChemiDoc MP system (Bio-Rad Laboratories). Image J software was used to analyze densitometric results of band.

### Plasmid construction

To construct a luciferase reporter vector, 3’-untranslated region (3’UTR) of Sirt4 was synthesized by PCR with the involvement of restriction enzyme cutting site. For sequence point mutation, site-directed mutagenesis of potential target site in the Sirt4 3’UTR was performed using a QuikChange Site-Directed Mutagenesis kit (Promega, Madison, WI, USA). The wild-type and mutant 3’UTR of Sirt4 were cloned into downstream of the luciferase open reading frame in the pMIR-report vector (Ambion, Carlsbad, CA, USA).

### Luciferase reporter assays

For luciferase assays, primary cardiomyocytes were infected recombinant lentivirus (MOI = 50) for 48 h, then transfected with 2 μg of the 3’UTR-luciferase reporter vector using Lipofectamine 2000 reagent (Thermo Fisher Scientific, Waltham, MA, USA) in 12-well plates. A quantity of 2 μg of pMIR vector plasmid was put into the well at the same time as a standard control. Cells were gathered after the 72 h-long incubation for the measurement of luciferase activities with the application of the Dual-Luciferase Reporter Assay System (Beyotime Biotechnology, Beijing, China) according to the manufacturer’s protocol.

### Statistical analysis

All experiment data were analyzed statistically by SPSS 13.0 software (SPSS, Inc, Chicago, IL, USA) and were expressed as mean ± standard deviation. Comparison of parameters between two groups was performed by Student’s t test (where distributions were normal) or Mann-Whitney U test (where distributions were significantly skewed). P<0.05 indicated that differences were statistically allowable.

## Results

### Down-regulation of miR-497 in cardiac hypertrophy

**Fig 1:**
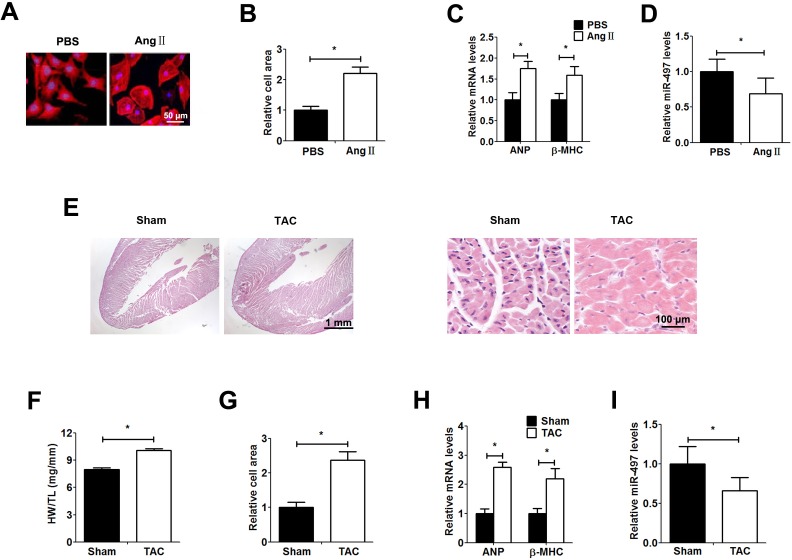
miR-497 is down-regulated in hypertrophic cardiomyocytes. **Establishment of hypertrophic cardiomyocytes treated with Ang II for 48h.** a: Immunofluorescence assay of α-actin was performed to identify cells. b: Relative cell area of cardiomyocytes detected by randomly measuring 10 cells from each section. c: ANP and β-MHC mRNA expression measured by qRT-PCR. d: miR-497 determined by qRT-PCR. e: Establishment of a mouse model of TAC-induced hypertrophy and histological analysis of hearts from different groups using H&E staining. f: The ratio of heart weight to tibial length. g: Relative cell area of cardiomyocytes detected by randomly measuring 10 cells from each section. h: ANP and β-MHC mRNA expression measured by qRT-PCR assay. i: miR-497 determined by qRT-PCR. Data are presented as the mean ± SD; *, P < 0.05, n = 5.

To identify the expression of miR-497 in cardiac hypertrophy, we established Ang Ⅱ-induced primary cardiomyocytes hypertrophy and TAC mice model. The levels of ANP and β-MHC were used to assess the extent of myocardial hypertrophy. The relative cell area increased significantly in response to the treatment with Ang Ⅱ (Fig 1A and 1B), and the expression levels of ANP and β-MHC were up-regulated obviously compared with control group (Fig 1C). Similarly, cardiac hypertrophy was further identified by the elevated ratio of heart weight to tibial length (HW/TL), the cell area and ANP and β-MHC levels in the TAC group (Fig 1E–1H). In addition, transthoracic echocardiography revealed that the interventricular septal end-diastolic thickness (IVSd), interventricular septal end-systolic thickness (IVSs), left ventricular posterior wall end-diastolic (LVPWd) and end-systolic thickness (LVPWs) markedly elevated in the TAC group compared with the sham group, while left ventricular end-diastolic dimension (LVIDd) and leftventricular end-systolic dimension (LVIDs) was significant reduced in TAC group. The fractional shortening (FS) and ejection fraction (EF) revealed no difference between the two groups (Table 1). The expression of miR-497 was significantly reduced in Ang Ⅱ-induced cardiomyocytes and TAC mice (Fig 1D and 1I).

**Table 1:** Cardiac structure and systolic function.

	Sham+NC	TAC+NC	TAC+miR-497
IVSd (10^−3^ cm)	149.67 ± 16.41	223.18 ± 30.10*	155.78 ± 16.03^#^
IVSs (10^−3^ cm)	221.75 ± 14.78	336.71 ± 36.57*	238.81 ± 23.57^#^
LVIDd (10^−3^ cm)	617.73 ± 35.81	512.34 ± 17.20*	621.98 ± 22.91^#^
LVIDs (10^−3^ cm)	405.38 ± 27.60	307.19 ± 23.46*	430.04 ± 26.08^#^
LVPWd (10^−3^ cm)	149.86 ± 8.94	210.73 ± 17.94*	155.48 ± 13.83^#^
LVPWs (10^−3^ cm)	183.41 ± 16.37	290.32 ± 29.02*	189.68 ± 18.51^#^
FS (%)	36.06 ± 4.88	38.44 ± 6.09	39.56 ± 5.02
EF (%)	68.83 ± 10.43	70.46 ± 13.18	69.08 ± 10.83

Values are represented as the mean ± SD

*, P < 0.05 versus sham+NC group

^#^, P < 0.05 versus TAC+NC group; n = 5.

### miR-497 suppresses cardiac hypertrophy in vitro and in vivo

**Fig 2:**
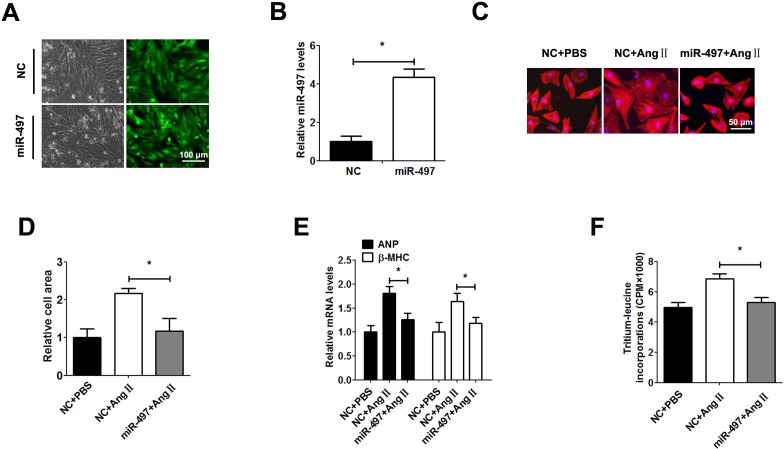
miR-497 suppresses cardiac hypertrophy in vitro. **Ang II-induced hypertrophic cardiomyocytes were infected with Leti-miR-497 or Leti-NC (MOI = 50) for 48 h.** a: Representative image of GFP in cardiomyocytes under a fluorescence microscope. b: miR-497 determined by qRT-PCR. c: Representative immunofluorescence image of α-actin staining. d: Relative cell area in Ang II-induced hypertrophic cardiomyocytes after infection. e: ANP and β-MHC mRNA expressions measured by qRT-PCR. f: Tritium-leucine incorporations detected by liquid scintillation counting. Data are presented as the mean ± SD; *, P < 0.05, n = 5.

**Fig 3:**
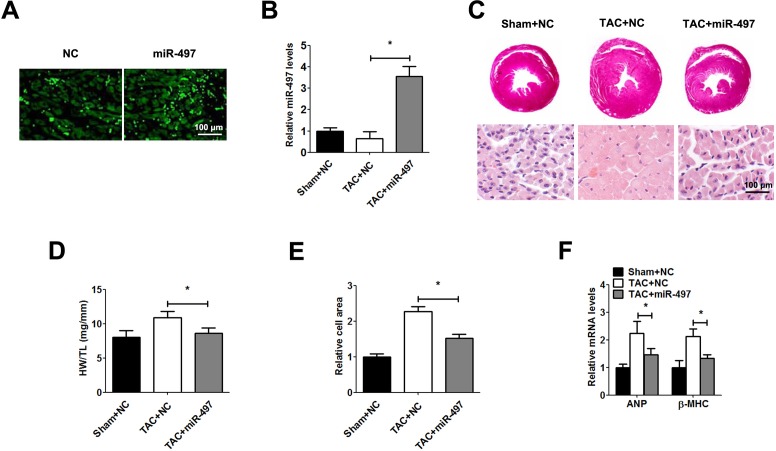
miR-497 suppresses cardiac hypertrophy in vivo. **Mice were subject to chest reopening injection with Leti-miR-497 or Leti-NC (3.5×10**^**7**^ **viral particles per mice).** a: Representative image of GFP in myocardium under a fluorescence microscope. b: miR-497 determined by qRT-PCR. c: Morphologic and histological analysis of hearts from different groups using H&E staining. d: The ratio of heart weight to tibial length. e: Relative cell area of cardiomyocytes detected by randomly measuring 10 cells from each section. f: ANP and β-MHC mRNA expression measured by qRT-PCR. Data are shown as the mean ± SD; *, P < 0.05, n = 5.

To explore the role of miR-497 in cardiac hypertrophy, we generated a miR-497-overexpressing recombinant lentivirus (Leti-miR-497). 48 h after infection, the level of miR-497 was significantly enhanced in cardiomyocytes (Fig 2A and 2B). Overexpression of miR-497 obviously inhibited Ang Ⅱ-induced cardiomyocytes hypertrophy (cell area, ANP and β-MHC levels) in Leti-miR-497 group compared with control group (Fig 2C–2E). Cardiomyocyte protein synthesis was determined by tritium-leucine incorporation. The tritium-leucine incorporation assay showed that overexpression of miR-497 reversed Ang Ⅱ-induced cardiomyocytes protein synthesis (Fig 2F). Similarly, in order to identify the role of miR-497 in cardiac hypertrophy in vivo, we infected Leti-miR-497 and control adenovirus in TAC mice. 48 h after infection, the level of miR-497 was significantly enhanced in cardiomyocytes of TAC mice (Fig 3A and 3B). Our result showed that cardiac hypertrophy (ratio of HW/TL, and cell area, ANP and β-MHC levels) was observably reversed after being infected with Leti-miR-497 (Fig 3C–3F). Transthoracic echocardiography demonstrated that overexpression of miR-497 decreased IVSd, IVSs, LVPWd and LVPWs, and increased LVIDd and LVIDs in TAC mice. As expected, EF% and FS% remained normal (Table 1).

### miR-497 inhibits Sirt4 expression by binding to its 3’UTR

**Fig 4:**
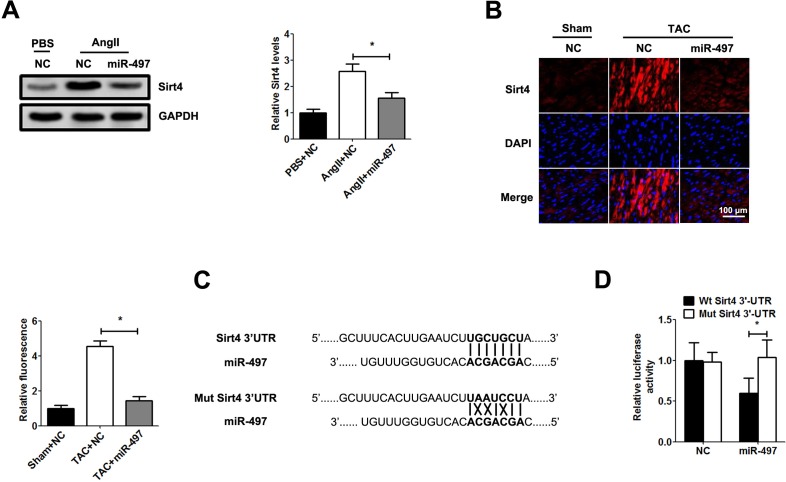
miR-497 negatively regulates Sirt4 expression. a: The expression of Sirt4 was detected by western blot in primary cardiomyocytes after infection with Leti-miR-497 or Leti-NC (MOI = 50) for 48 h. b: Immunofluorescence assay of Sirt4 expression in cardiac tissues after infection with Leti-miR-497 or Leti-NC (3.5×10^7^ viral particles per mice). c: The potential binding sequence in 3’UTR of Sirt4 and a mutant sequence were cloned into the pMIR luciferase vector. d: Primary cardiomyocytes were transfected reporter plasmid and were infected Leti-miR-497 for 48 h. Relative luciferase intensity was determined with a luciferase assay. Data are presented as the mean ± SD; *, P < 0.05, n = 5.

Then, we investigated the molecular mechanism which miR-497 inhibited the cardiac hypertrophy. By scanning the target gene of miR-497 with bioinformatics method, we found that Sirt4 was a potential target gene of miR-497. To confirm the correlation between miR-497 and Sirt4, we detected the expression of Sirt4 in hypertrophic cardiomyocytes. As expected, immunofluorescence and western blot demonstrated that the expression of Sirt4 was significantly enhanced in Ang Ⅱ-induced cardiomyocytes and TAC mice, and the expression of Sirt4 was significantly down-regulated with the overexpression of miR-497 both in vitro and in vivo (Fig 4A and 4B). Furthermore, to clarify whether miR-497 suppress the expression of Sirt4 directly or not, we investigated the possible miR-497 seed sequence in the 3’UTR of Sirt4 using the TargetScan algorithms and cloned the 3’UTR of wild-type and mutant Sirt4 into the luciferase reporter gene system (Fig 4C). The activity of the luciferase reporter gene linked to the 3’UTR of wild-type Sirt4 was reduced with the presence of miR-497, while that of mutant Sirt4 did not change (Fig 4D).

## Discussion

Irreversible pathological cardiac hypertrophy is one of the main promoters to heart diseases, and aberrant expression of miRNAs is reported to be a powerful factor in the process of pathological cardiac hypertrophy [11–13]. Growing studies demonstrate that miRNAs play an important role in the progression of cardiac hypertrophy and even heart failure [14–16]. Recently, miR-497 is up-regulated in cardiac hypertrophy and heart failure and is identified as a novel regulator of cardiac hypertrophy [9]. However, its particular molecular mechanism needs further research.

In this study, our result demonstrated that miR-497 was down-regulated obviously in cardiac hypertrophy, suggesting that miR-497 was involved in the development cardiac hypertrophy. Overexpression of miR-497 reduced the cell area and improved cardiac hypertrophy, which indicated that miR-497 suppressed cardiac hypertrophy morphologically. Given that the hypertrophic extent of cardiacmyocytes is assessed by the increase of protein synthesis and gene transcription, the significant reduction of tritium-leucine incorporation indicated that the protein synthesis of hypertrophic cardiomyocytes was inhibited as well. Consistently, overexpression of miR-497 in TAC mice revealed the parallel results, accompanied by decreased cell area morphologically and reduction of ANP and β-MHC expressions. These results suggested that miR-497 could suppress cardiac hypertrophy in the overload-induced hypertrophic cardiomyocytes. Importantly, unchanged EF% and FS% indicated that miR-497 didn’t affect cardiac function, and it suppressed cardiac hypertrophy in the condition of compensatory hypertrophy. Our study indicated that miR-497 was a critical regulator in hypertrophic cardiomyocytes in vitro and in vivo. Interestingly, another report demonstrates that miR-497 is higher expressed in adult mice heart than in other tissues including lung, kidney, liver and brain, and overexpression of miR-497 enhances apoptosis and autophagy and inhibits cell survival in cultured neonatal cardiomyocytes. In contrast, silencing of endogenous miR-497 provides protection against IR-induced cardiomyocyte death and apoptosis by targeting Bcl-2 and LC3B [17]. In our study, miR-497 suppressed Ang Ⅱ and TAC-induced cardiac hypertrophy, and also plays a protective role in cardial disorder. From this point, miR-497 may act as a conducive roles in various pathophysiological process in cardiomyocyte and exert different effects responded to various myocardial damages.

Then we performed further study to investigate the molecular mechanism behind the correlation of miR-497 and Sirt4. There was a reverse relationship between miR-497 and Sirrt4 expression in cardiomyocytes. In addition, we also confirmed that miR-497 suppress cardiac hypertrophy by targeting 3’UTR of Sirt4 during cardiac hypertrophy. Sirt4, as a mitochondrial NAD+-depending ADP-ribosyltransferase, regulates cellar metabolism functions [18–19]. It is proposed that Sirt4 can meditate adaptive responses to cellular environment and plays a significant role in regulating oxidative stress [20–21]. A previous study reported that Sirt4 can promote the cardiac hypertrophy by up-regulating the reactive oxygen species (ROS) levels in Ang Ⅱ-induced hypertrophic cardiomyocytes [20]. In our future work, we will focus on whether miR-497 regulates ROS by targeting Sirt4 in cardiac hypertrophy.

For the first time, we provide a convictive evidence that miRNA-497 is negatively correlated to Sirt4 and play a key role in suppressing cardiac hypertrophy by targeting the 3’UTR of Sirt4. We confirm that miR-497 is a possible therapy target in improving cardiac hypertrophy.

## Supporting Information

S1 File Raw data. (XLSX)
